# Anti-TNF therapy: what have we learned in 12 years?

**DOI:** 10.1186/1478-6354-13-S1-S1

**Published:** 2011-05-25

**Authors:** Joachim R Kalden

**Affiliations:** 1 Nikolaus-Fiebiger-Zentrum, Glueckstrasse 6, 91054 Erlangen, Germany

## 

Two decades ago, the available treatment options were not effective for many persons with rheumatoid arthritis (RA) and other inflammatory rheumatic diseases. At this time RA patients had a significantly decreased life expectancy due to a relatively incontrolled inflammatory activity of RA combined with an increased incidence of comorbidities and severe treatment-related side effects. After 5 years of disease duration, a considerable percentage of patients became unemployed. The available disease-modifying antirheumatic drugs were able to slow disease progression, but they could not stop it. This unsatisfying situation changed significantly at the beginning of the 1990s, when treatment options for RA patients expanded to include TNF blockers. These blockers were shown to significantly decrease not only the inflammatory activity of RA, but also the radiographic progression. This breakthrough in therapeutic options was fostered by the pioneering research of Prof. Sir Ravinder Maini and Prof. Sir Marc Feldman working at the Kennedy Institute in London, who introduced a chimeric monoclonal antibody against TNFα in the treatment of RA patients.

Since these early clinical trials, TNF inhibitors have radically changed the entire therapeutic approach, not only for RA but also for ankylosing spondylitis (AS) and for psoriatic arthritis (PsA). While the focus once was symptom mitigation, rheumatologists now seek control of disease progression. Today, TNF inhibitors effectively suppress and control the inflammation driving these diseases, and thereby prevent irreversible tissue damage and disability. Radiographic studies are showing that progressive joint damage can be halted under certain conditions in some patients. These conditions and patients are currently being elucidated. New frontiers include early diagnosis and referral; optimizing treatment regimens; identifying patients at risk of rapidly progressing disease; and instituting early, rapid TNF blockade in these patients. Using combination therapy with TNF inhibitors and disease-modifying antirheumatic drugs – specifically methotrexate – at an early stage of RA, disease progression can be stopped or at least significantly retarded.

The four articles in this supplement present up-to-date information, current data, and the best thinking on anti-TNF therapy in RA, AS, and PsA. Since research over the past decade revealed that these three disorders share an inflammatory mechanism fueled especially by TNF, efforts to produce viable TNF inhibitors have accelerated.

Josef S Smolen and Paul Emery, in their article ‘Infliximab: 12 Years of Experience’, expound upon the record of efficacy and safety amassed for this TNF inhibitor. Research on infliximab, which began with the first randomized controlled study in a rheumatic disease, stimulated most of the development of TNF neutralizing therapies. The authors summarize the major trials in RA, AS, and PsA that contributed to this body of evidence.

In the second article, ‘Understanding Emerging Treatment Paradigms in Rheumatoid Arthritis’, Ferdinand C Breedveld and Bernard Combe assert that most patients with RA are undertreated. They outline accumulating data showing that intensive treatment in the early stages of RA can slow or stop disease progression, and they describe alternatives to traditional step-up approaches and to sequential monotherapy with disease-modifying antirheumatic drugs.

Next, Georg Schett and colleagues focus on bone and cartilage injury in all three diseases, with an emphasis on key knowledge arising from TNF research and how it has paved the way for future advances. Their article, ‘Structural Damage in Rheumatoid Arthritis, Psoriatic Arthritis, and Ankylosing Spondylitis: Traditional Views, Novel Insights Gained from TNF Blockade, and Concepts for the Future’, explores the different pathologies as well as radiographic data for the interaction between inflammation and structural progression.

Lastly, Paul P Tak and I discuss innovative approaches – with TNF inhibitors but also with other biologic agents – to inflammatory arthritides, including new targets within the inflammatory cascade, agents designed to affect associated pathways, and improved management strategies involving early diagnosis and referral. In the article ‘Advances in Rheumatology: New Targeted Therapeutics’, we explore the variable response to targeted therapy and consider how best to integrate these advanced therapies into daily practice.

Our goal with the present supplement is to provide a valuable, current resource from which additional dialog may spring. We all agree that optimal treatment, initiated sooner, may reduce costs and risks to patients, and therefore achieve our common ambition – to help the patient and to attain low disease activity or remission in persons with inflammatory arthritis.

## Abbreviations

AS: ankylosing spondylitis; PsA: psoriatic arthritis; RA: rheumatoid arthritis; TNF: tumor necrosis factor.

## Competing interests

The author declares that he has no competing interests.

